# The efficacy and safety of direct superior approach (DSA) for total hip arthroplasty: a systematic review and meta-analysis

**DOI:** 10.1186/s13018-023-04233-2

**Published:** 2023-10-10

**Authors:** Zhuangzhuang Zhang, Fukang Zhang, Xin Yang, Hua Fan, Qinghao Cheng, Hongzhang Guo

**Affiliations:** 1 The First Clinical Medical College of Gansu University of Chinese Medicine (Gansu Provincial Hospital), Lanzhou, China; 2 Orthopedics IV, Gansu Provincial Hospital, No. 204 Donggang West Road, Chengguan District, Lanzhou, China

**Keywords:** Direct superior approach, Minimally invasive, Hip replacement, Mate analysis, Systematic review

## Abstract

**Objective:**

To systematically evaluate the clinical safety and efficacy of the direct superior approach and the conventional surgical approach.

**Date sources:**

From PubMed, Embase, the Cochrane Library, and China Knowledge Network up to January 30, 2023.

**Main results:**

A total of 7 case series involving 4306 patients undergoing total hip arthroplasty were included, including 679 patients with direct superior approach. All outcome measures: Oxford Hip Score [MD = 0.30, 95% CI (− 0.87, 1.47), *P* = 0.62], Hip Harris Score [MD = − 0.18, 95% CI (− 0.86, 0.49), *P* = 0.59], intraoperative blood loss [MD = − 54.14, 95% CI (− 102.75,-5.52), *P* = 0.03], transfusion rate [MD = 0.49, 95% CI (0.29, 0.83), *P* = 0.008], Limb Length Differences [MD = − 0.21, 95% CI (0.02, 0.39), *P* = 0.03], Length of Stay [MD = − 0.61, 95% CI (− 0.69, − 0.52), *P* < 0.00001].

**Conclusions:**

The DSA was superior to conventional access in terms of incision length, bleeding, postoperative transfusion rate, and early postoperative HHS. In addition, our study found that because the DSA has less tissue damage, it has the potential advantages of accelerating patient recovery after surgery, shortening hospitalization time, and reducing patient economic pressure, which can significantly improve patient quality of life and satisfaction.

## Introduction

With the continuous increase of the world's elderly population, the incidence curve of hip joint diseases and osteoporosis is also on the rise [1]. In 2000, Cooper estimated that 1.6 million hip fractures occurred among the 9 million patients with osteoporotic fractures worldwide, and the number of hip fractures worldwide will increase from 1.6 million in 2000 to 6.3 million in 2050 [2]. Artificial hip replacement has become an effective treatment for end-stage hip diseases such as osteoarthritis and femoral head necrosis and femoral neck fractures in clinical practice, and its main purpose is to restore and improve joint motion function, relieve joint pain, and correct deformities [3]. More than 1 million total hip replacements are currently performed annually worldwide, and the number of total hip replacements continues to increase due to the current global aging (increase in life expectancy of the global population) and the increase in the global obese population [4]. To improve early recovery, the choice of total hip arthroplasty approach has also received increasing attention, such as the posterolateral approach and the posterolateral approach. Among them, the posterolateral approach is the most common approach for hip replacement because it provides good visualization of the acetabulum and femur for both initial and revision hip arthroplasty [5]. However, the risk of trauma, blood loss, and postoperative complications is higher with the traditional surgical approach mentioned in the published literature [6], whereas the minimally invasive surgical approach can improve surgical outcomes and theoretically reduce surgical medically induced injuries compared to the traditional approach, and more and more orthopedic surgeons and researchers are proposing the development of minimally invasive techniques as a future trend in surgical treatment [7].

Direct Superior Approach (DSA) is a new minimally invasive technique that has been applied in recent years. It is a muscle-sparing approach for hip joint replacement, similar to the posterior approach. It was first proposed by Stephen Murphy in 1999 and requires specific instruments to preserve the external rotator muscles' tendons. The approach involves blunt dissection of the gluteus maximus and opening the upper joint capsule to reach the femoral neck [8]. As a minimally invasive technique, the DSA is theoretically advantageous over traditional surgical methods for faster postoperative recovery. The DSA is favored and recognized by surgeons due to its small incision and minimal soft tissue damage, resulting in faster postoperative recovery. However, there is currently no consensus on the specific surgical efficacy and safety of the DSA. Michele and Jacopo et al. [9] showed that the DSA group had less intraoperative blood loss as well as better early gait recovery, but the DSA group had a longer operative time. Although several scholars have studied the DSA in recent years, a large portion of previous studies conducted research with limited patient numbers and drew uncertain conclusions on these questions. Therefore, these studies were pooled and a systematic review and meta-analysis was conducted to establish more conclusive results based on a larger patient population, regarding the efficacy and safety of DSA total hip arthroplasty in older people, and future complications in the follow-up, and to provide sufficient evidence-based medical evidence for the clinical application of the DSA.

## Methods

The protocol for this systematic review was registered in the PROSPERO database (CRD42023401009).

### Inclusion and exclusion criteria

#### Inclusion criteria

Only cohort studies and randomized controlled studies were included. (1) confirmed diagnosis of degenerative hip arthritis, ischemic necrosis of the femoral head or femoral neck fracture; (2) need for artificial total hip replacement; (3) the first artificial total hip replacement; (4) the DSA and conventional approach (posterior approach, posterior lateral approach); (5) outcome indicators reported at least one of the following: operating time, incision length, blood loss, blood transfusion rate, Length of Stay (LOS), lower leg discrepancy (LLD), Harris hip score (HHS), Oxford hip sore (OHS).

#### Exclusion criteria

(1) Duplicate publications; (2) Inability to extract basic data, lack of original data, or incomplete data; (3) Lack of comparison between two methods; (4) Literature reviews, Case reports, Theoretical explorations, Simple experimental studies, and Empirical summaries.

### Search strategy

Computer searches of relevant studies from PubMed, Embase, the Cochrane Library, and China Knowledge Network (CNKI) were conducted from build to January 30, 2023, to identify eligible trials and studies on DSA. The Cochrane Handbook was used for all aspects of the international systematic reviews, and studies were prepared according to the Preferred Reporting Items in Systematic Reviews and Mate Analysis (PRISMA) statement [10]. The search strategy is shown in Table 1. The keywords for the Chinese search were direct superior approach, total hip arthroplasty; the keywords for the English search were Direct Superior Approach, DSA, total hip arthroplasty, total hip replacement.

**Table 1:** Search strategy

Cochrane Library	Embase	PubMed
1. MeSH descriptor: [Arthroplasty, Replacement, Hip] explode all trees	1. [Arthroplasty, Replacement, Hip]:exp	1. "Arthroplasty, Replacement, Hip"[Mesh]
2. (Hip Prosthesis Implantation):ti,ab,kw	2. (Hip Prosthesis Implantation):ti,ab,kw	2. “Hip Prosthesis Implantation “[Title/Abstract]
3. (Hip Prosthesis Implantations):ti,ab,kw	3. (Hip Prosthesis Implantations):ti,ab,kw	3. “Hip Prosthesis Implantations “[Title/Abstract]
4. (Hip Replacement Arthroplasty):ti,ab,kw	4. (Hip Replacement Arthroplasty):ti,ab,kw	4. “Hip Replacement Arthroplasty” [Title/Abstract]
5. (Hip Replacement Arthroplasties):ti,ab,kw	5. (Hip Replacement Arthroplasties):ti,ab,kw	5. “Hip Replacement Arthroplasties” [Title/Abstract]
6. (Total Hip Replacement):ti,ab,kw	6. (Total Hip Replacement):ti,ab,kw	6. “Total Hip Replacement” [Title/Abstract]
7. (Total Hip Arthroplasty):ti,ab,kw	7. (Total Hip Arthroplasty):ti,ab,kw	7. “Total Hip Arthroplasty” [Title/Abstract]
8. 1 OR 2 OR 3 OR 4 OR 5 OR 6 OR 7	8. 1 OR 2 OR 3 OR 4 OR 5 OR 6 OR 7	8. 1 OR 2 OR 3 OR 4 OR 5 OR 6 OR 7
9. (Direct superior approach):ti,ab,kw	9. (Direct superior approach):ti,ab,kw	9. “Direct superior approach” [Title/Abstract]
10. (DSA):ti,ab,kw	10. (DSA):ti,ab,kw	10. “DSA” [Title/Abstract]
11. 9 OR 10	11. 9 OR 10	11. 9 OR 10
12. 8 AND 11	12. 8 AND 11	12. 8 AND 11
13. Limit 12 to English language	13. Limit 12 to English language	13. Limit 12 to English language
Results 29	Results 30	Results 29
Date January 30, 2023	Date January 30, 2023	Date January 30, 2023

### Literature quality evaluation and data extraction

The two researchers (Zhang Zhuangzhuang and Zhang Fukang) independently evaluated the quality and extracted data according to the inclusion criteria before cross-checking. If there are any differences between them, a senior researcher (Guo Hongzhang) will make a decision. Randomized controlled trials were evaluated using the Quality Cochran Risk Assessment Tool [11]. The Newcastle Ottawa Scale (NOS) [12] assessed the quality of the cohort study, which consisted of three main components: selection of study groups, comparability between groups, and determination of exposure results. The total score is 9 points, and ≤ 5 points are considered to be a high-risk bias; A general score ≥ 7 is considered a low-risk bias. The following information was extracted from the study: (1) Characteristics of the study (first author, publication time, surgical modalities, study type, case characteristics, etc.); (2) Results indicators: operating time, incision length, blood loss volume, blood transfusions, LOS, LLD, OHS, and HHS.

### Statistical analysis

After data extraction from the original study, data analysis was performed using RevMan (Review Manager Version 5.3, The Cochrane Collaboration, 2014). Dichotomous variables were expressed by odds ratio (OR) and 95% confidence interval (CI), and continuous variables were expressed by mean difference (MD) and 95% CI. Statistical* I*^2^ quantitatively evaluated the magnitude of heterogeneity among the included studies. When *I*^2^ was < 50%, suggesting less heterogeneity, the statistical analysis was conducted using a fixed effect model (FEM); if *I*^2^ was > 50%, suggesting greater heterogeneity, a random effect model (REM) was used. Sources of heterogeneity were further analyzed, and apparent clinical heterogeneity was addressed using methods such as subgroup analysis or sensitivity analysis. Subgroup analysis was performed depending on the duration of OHS follow-up in the included studies. When the outcome indicator under study was reported in only 1 study, only descriptive analyses were performed.

## Results

### Literature search results

**Fig. 1:**
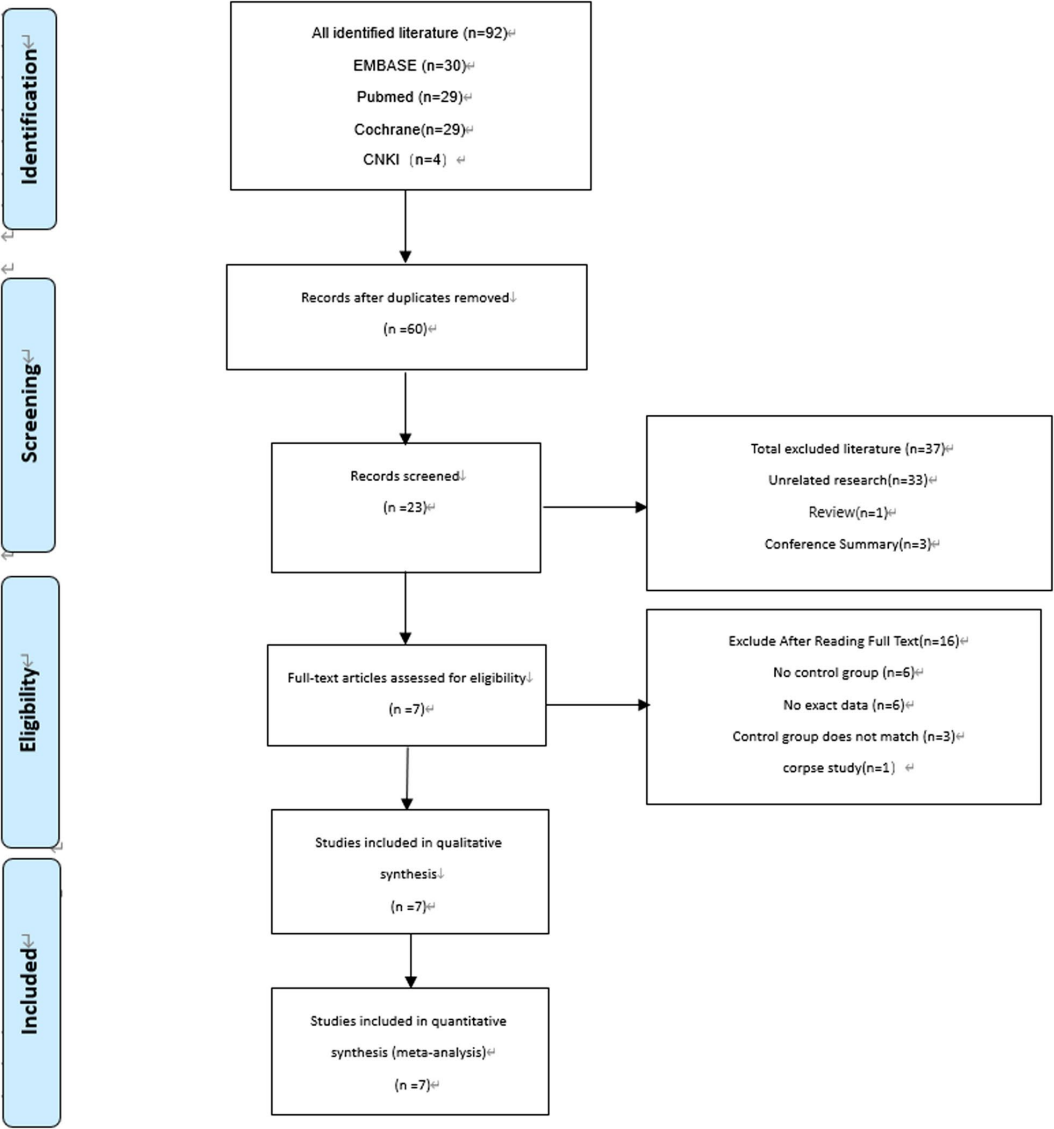
Flow chart of literature screening

Initially, 92 studies were obtained, of which 32 duplicate studies were excluded. 37 studies were excluded due to review comments, conference abstracts, and irrelevant research after reading the titles and abstracts. 6 studies were excluded due to the lack of a control group. Further examination of the context excluded 3 studies that lacked necessary data and 3 studies without data. 3 studies that did not meet the inclusion criteria were excluded. In addition, 1 study that focused on corpses was excluded, and ultimately 7 studies that met the criteria were selected. The process of inclusion and exclusion of literature is shown in Fig. 1.

### Basic characteristics and quality evaluation

**Fig. 2:**
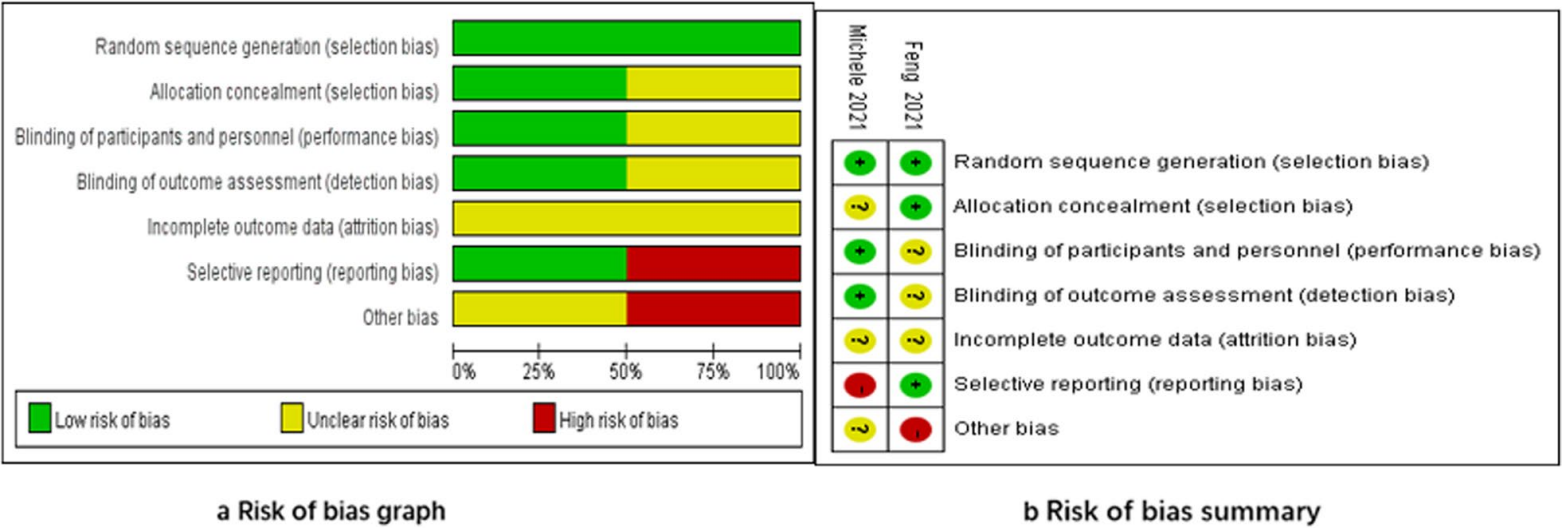
Risk of bias

This study included a total of 7 studies [9, 12, 17] with basic characteristics of 4306 patients as shown in Table 1. Regarding gender ratio, their studies [13–15] did not mention it. Regarding body mass index, one study [15] did not mention it. By comparing the baseline characteristics such as age and gender of patients in each included study, it was found that there was no statistically significant difference between the two groups of patients. The studies were comparable (*P* > 0.05). A summary of basic characteristics is shown in Table 2. Two randomized controlled studies [9, 12] were included in this research paper. Due to the specificity of the procedure, all randomized controlled trials did not report the method of participant-operator combination. In addition, the two RCTs [9, 12] included explained randomization, but did not mention a randomized paired design. Risk bias was performed for the two RCTs [9, 12] included (Fig. 2). As shown in Table 3, the NOS scores of the 5 cohort studies [13–17] were 6, 7, 7, 7, and 6, respectively. Overall, the 7 studies included in this research had good quality, with standardized research design and good quality**.**

**Table 2:** Study characteristics of all studies

Inclusion instudies	Type of study	Country	Sample sizes		Ages		Gender (F/M)		BMI	
			S	C	S	C	S	C	S	C
Michele [9]	RCT	Italy	22	23	74 ± 8.9	72 ± 7.7	7/5	10/13	23 ± 2.8	24 ± 2.0
Feng [13]	RCT	China	30	30	47.3 ± 14.1	53.7 ± 18.7	NR	NR	23.2 ± 3.3	22.9 ± 3.5
Leonard [14]	CR	England	100	100	68	69.05	39/61	39/61	28	28.9
Eustathios [15]	CR	Greece	100	100	65.39 ± 8.38	65.51 ± 7.85	NR	NR	28.38 ± 3.09	27.94 ± 2.98
Denis [16]	CR	America	42	196	49.9 ± 7.1	63.9 ± 6.1	NR	NR	NR	NR
Matthew [16]	CR	America	333	3162	62 ± 11	64 ± 11	46.2/53.8	43.1/56.9	28.8 ± 5.3	30.2 ± 6.2
Bouke [18]	CR	Netherlands	52	52	69 ± 8.4	69 ± 8.4	24/28	18/34	25 ± 3.4	30.2 ± 6.2

**Table 3:** The Newcastle–Ottawa-scale of non-randomized controlled studies

Inclusion in studies	Selection of study subjects				Comparability between groups	Outcome measures			Scores
	1	2	3	4		a	b	c	
Leonard [14]	*	*	*	*		*	*		6*
Eustathios [15]	*	*	*	*	*	*	*		7*
Denis [16]	*	*	*	*	*	*	*		7*
Matthew [17]	*	*	*	*	*	*	*		7*
Bouke [18]	*	*	*	*		*	*		6*

^‡^: 1. Representation of exposed groups, 2. Selection of non-exposed queues, 3. Determination of exposure factors, 4. No outcome indicators to observe at the start of the study, a. Evaluation of closing indicators, b. Adequate follow-up time(Follow-up time ≥ 5 years.), c. Completeness of follow-up in the exposed and non-exposed groups

## Mate results

### Main results

#### Oxford hip score (OHS)

**Fig. 3:**
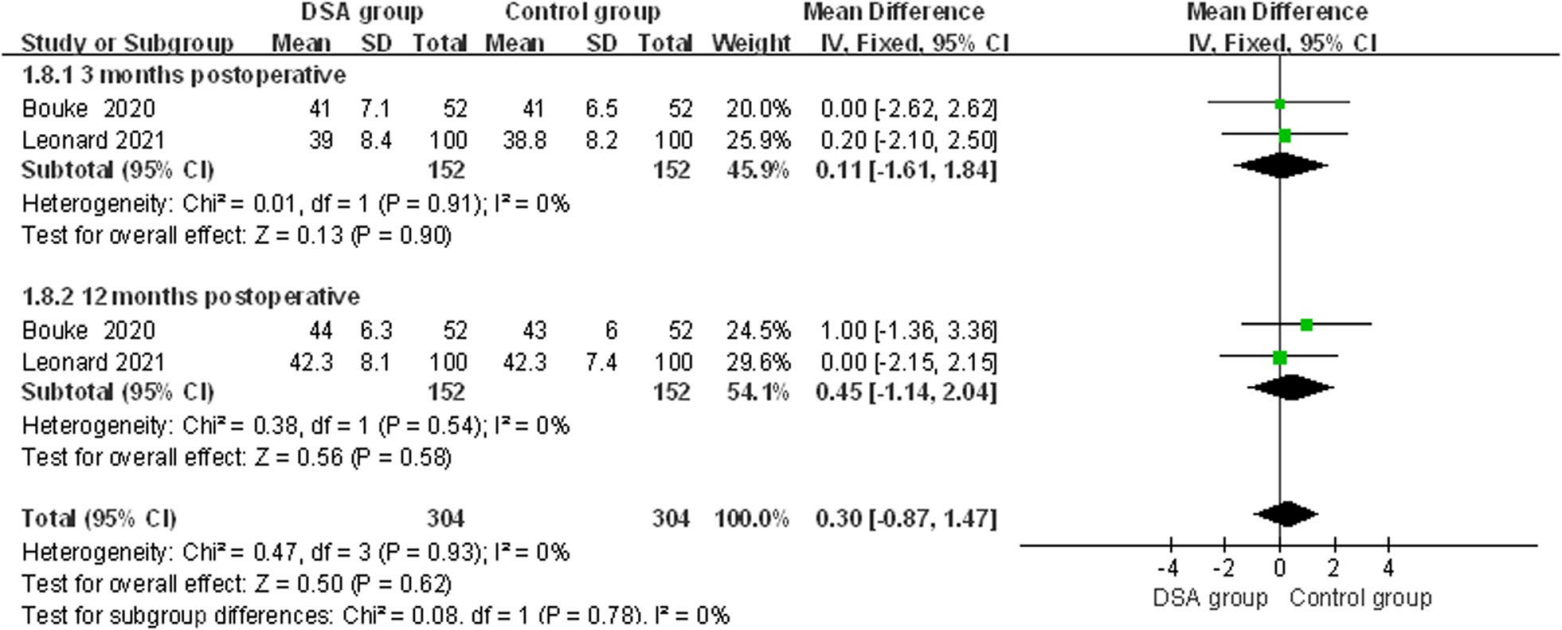
Forest plot of OHS

Two studies [14, 18] recorded postoperative Oxford hip scores, which did not differ significantly due to the heterogeneity of the results (*x*^2^ = 0.47, d*f* = 3, *I*^2^ = 0%, *P* = 0.93). Therefore, a fixed-effects model was used for Mate analysis. The results are shown in Fig. 3, there was no significant difference between the two groups [MD = 0.30, 95% CI (− 0.87, 1.47), *P* = 0.62], and there was no difference between the DSA and the conventional approach for OHS.

Subgroup analysis based on follow-up time, subgroup analysis of Oxford hip scores at 3 months and 1 year postoperatively showed no significant difference between the two groups at 3 months [MD = 0.11, 95% CI (− 1.61, 1.84), *P* = 0.90] and 1 year postoperatively [MD = 0.45, 95% CI (− 1.14, 2.04), *P* = 0.58] (Fig. 3).

#### Hip Harris score (HHS)

**Fig. 4:**
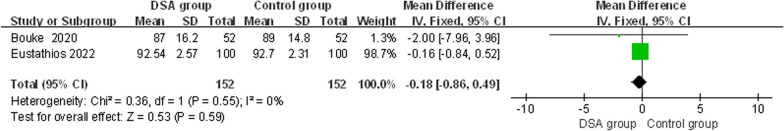
Forest plot of HHS

Two studies [15, 18] reported postoperative HHS scores, but there was no significant difference in the heterogeneity of the results [*x*^2^ = 0.36, d*f* = 1, *I*^2^ = 0%, *P* = 0.55]. Therefore, a fixed-effect model was used for the meta-analysis. As shown in Fig. 4, there was no significant difference between the two groups [MD = − 0.18, 95% CI (− 0.86, 0.49), *P* = 0.59] (Fig. 4), indicating no significant difference in HHS between the DSA and the traditional approach.

#### Intraoperative blood loss

**Fig. 5:**
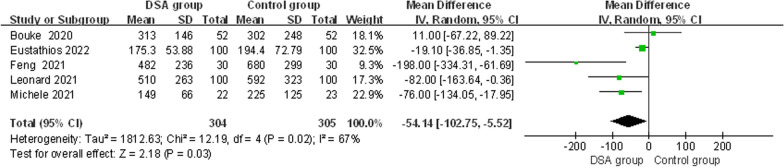
Forest plot of intraoperative blood loss

Five studies [9, 13–15, 18] reported intraoperative blood loss. Due to significant heterogeneity in the results (*x*^2^ = 12.19, d*f* = 4, *I*^2^ = 67%, *P* = 0.02), a random-effects model was used for meta-analysis. As shown in the figure, the intraoperative blood loss in total hip arthroplasty with the DSA was lower than that in the control group, and the result was statistically significant [MD = − 54.14, 95% CI (− 102.75, − 5.52), *P* = 0.03] (Fig. 5). There was a difference in intraoperative blood loss between the DSA and traditional approaches.

#### Blood transfusion rate

**Fig. 6:**
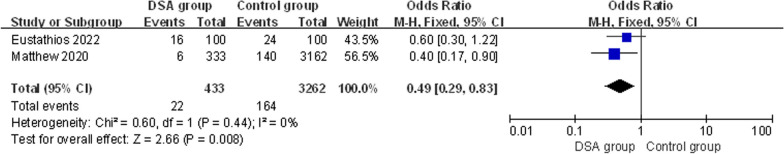
Forest plot of blood transfusion rate

Two studies [15, 17] reported the rate of blood transfusion during surgery, and there was no significant difference in the heterogeneity of the results (*x*^2^ = 0.6, d*f* = 1, *I*^2^ = 0%, *P* = 0.44). Therefore, a fixed-effect model was used for the Mate analysis. The results showed that the blood transfusion rate for total hip arthroplasty via the DSA was lower than that of the control group, and the results were statistically significant [OR = 0.49, 95% CI (0.29, 0.83), *P* = 0.008] (Fig. 6). There was a difference in the transfusion rate between the DSA and the traditional approach.

#### Limb length differences

**Fig. 7:**
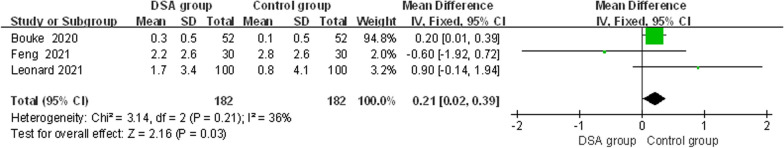
Forest plot of limb length differences

Three studies [13, 14, 18] reported the difference in length of bilateral lower limbs after surgery, but due to heterogeneity of the results, there was no significant difference (*x*^2^ = 3.14, d*f* = 2, *I*^2^ = 36%, *P* = 0.21). Therefore, a fixed-effect model was used for Mate analysis. The results showed that the control group was better than the DSA group, with statistical significance [MD = − 0.21, 95% CI (0.02, 0.39), *P* = 0.03] (Fig. 7), and there was a difference in the length of bilateral lower limbs between the DSA and the traditional approach.

#### Length of stay

**Fig. 8:**
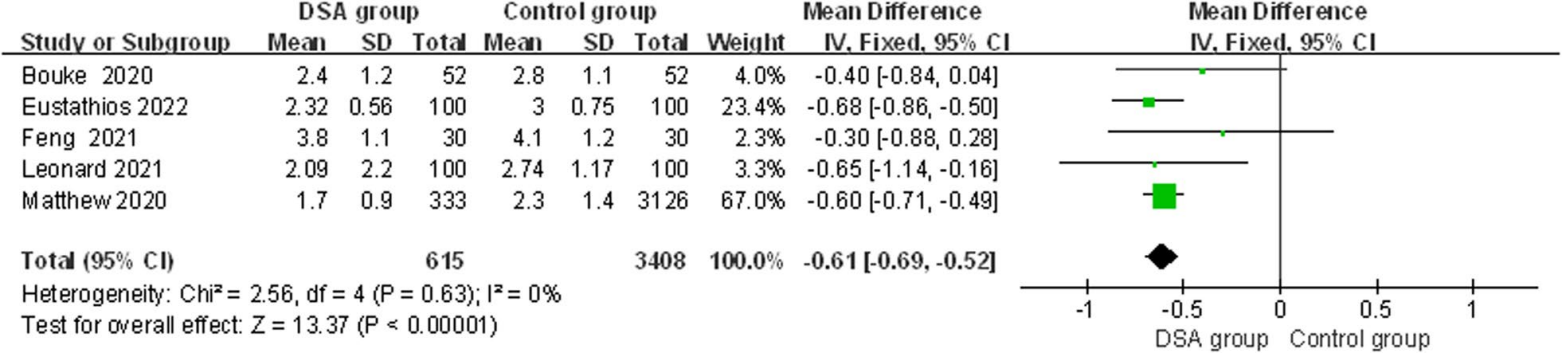
Forest plot of length of stay

Five studies [13–15, 17, 18] recorded the duration of surgical hospitalization, and because of the significant heterogeneity of the results (*x*^2^ = 2.56, d*f* = 4, *I*^2^ = 0%, *P* = 0.63). Therefore, a fixed-effect model was used for Mate analysis. The results showed that the length of stay for total hip arthroplasty via the DSA was lower than that of the control group [MD = − 0.61, 95% CI (− 0.69, − 0.52), *P* < 0.00001] (Fig. 8).In this study, five studies reported postoperative hospital stays and we performed Mate analysis, which was statistically significant in terms of postoperative hospital stay, and the DSA had a lower hospital stay than the control group.

### Secondary results

#### Operating time

**Fig. 9:**
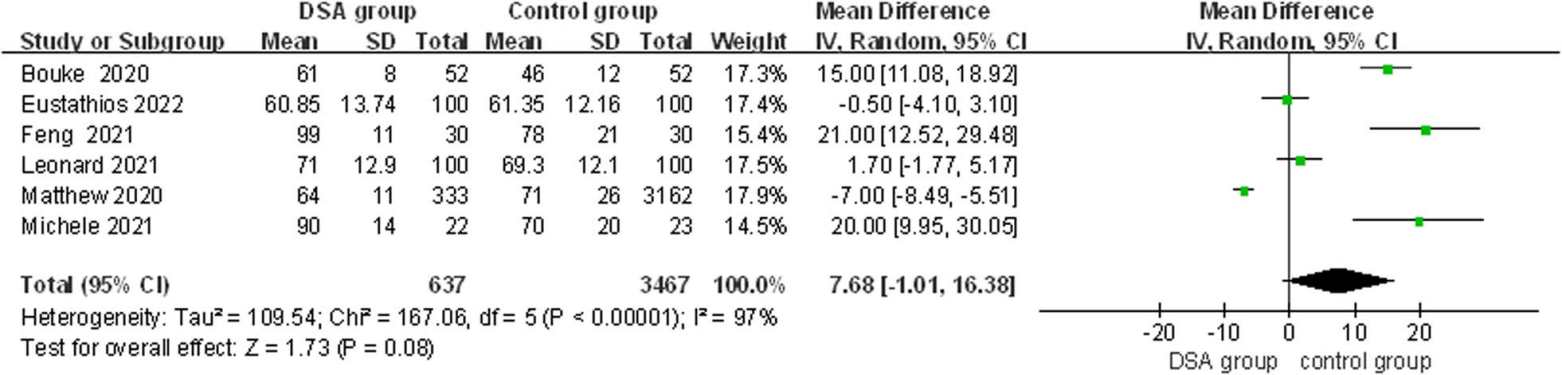
Forest plot of operating time

Six studies [9, 13–15, 17, 18] recorded the operation time. Due to the large heterogeneity of the results (*x*^2^ = 167.06, d*f* = 5, *I*^2^ = 97%, *P* < 0.00001), the random effect model was used for Mate analysis. The results showed that there was no significant difference between the two groups [MD = 7.68, 95% CI (− 1.01, 16.38), *P* = 0.08] (Fig. 9), and there was no difference in the operation time between the DSA and the traditional approach.

#### Incision length

**Fig. 10:**
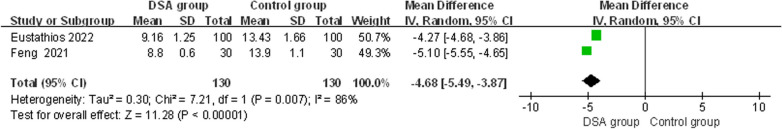
Forest plot of incision length

Two studies [13, 15] reported the length of surgical incisions. Due to significant heterogeneity in the results (*x*^2^ = 7.21, d*f* = 1, *I*^2^ = 86%, *P* = 0.007), a random-effects model was used for Mate analysis. As shown in the figure, the incision length of the DSA approach for total hip arthroplasty was superior to the control group, with statistical significance [MD = − 4.68, 95%CI (− 5.49, − 3.87), *P* < 0.00001] (Fig. 10). There was a difference in the surgical incision between the DSA and the traditional approach.

### Sensitivity analysis

Sensitivity analysis was performed on the data of DSA versus conventional access for total hip arthroplasty in terms of operative time and surgical incision. When one study was excluded individually and the MD values of the remaining studies were combined, the values of *I*^2^ did not change significantly and the results of the studies were relatively stable, indicating that the results of the Meta-analysis were reliable.

## Discussion

THA is currently an effective method for relieving pain, restoring joint function, and improving quality of life in patients with end-stage hip osteoarthritis, femoral neck fractures, and other conditions [19]. However, surgical pain remains a significant concern, with one of the important reasons being that different surgical approaches cause varying degrees of soft tissue damage during surgery [6]. In addition, the surgical approach for total hip arthroplasty has a significant impact on postoperative gait, hip stability, and muscle function.

Therefore, choosing the most appropriate surgical approach for total hip arthroplasty to minimize soft tissue damage, pain, and complications and to accelerate postoperative functional recovery is crucial and is currently a controversial focus of total hip arthroplasty [20, 21]. One important direction in the improvement of surgical techniques is the introduction of "small incisions" or "minimally invasive" approaches, with the advantages of reducing pain, decreasing anesthesia requirements, and accelerating recovery and functional restoration [22]. The DSA is a newly improved approach in recent years [7]. Compared with the traditional posterior and the posterior-lateral approaches, the DSA preserves the gluteus medius, iliotibial band, and external obturator tendon, causing less damage to the soft tissue of the hip joint, such as the gluteus minimus and piriformis, thus effectively reducing postoperative pain and dislocation. However, the DSA requires the use of specific hooks and instruments to facilitate the insertion of the femoral stem and acetabular component [23].

### Operating time

Excessive operative time prolongs the possible increase in intraoperative bleeding, which increases the probability of postoperative transfusion and leads to increased potential surgical risks for patients, such as the risk of allergic reaction to transfusion and infections from infectious diseases. In the results of this study, it was shown that in terms of operative time, the direct superior approach had a longer operative time, indicating an increased intraoperative risk of bleeding and infection with the DSA compared to the conventional approach. The mean operative time of the DSA was slightly longer than that of the conventional approach, and the reasons for this were analyzed: It may be due to the smaller skin incision, reduced surface soft tissue stripping, and limited surgical field of view, which prolonged the operative time [24], in addition, the incision suturing session was mostly done by the junior surgeon at the end of the procedure, which may affect the overall operative time. In addition, the longer operative time for the DSA may be closely related to the learning curve. BabarKayani et al. [23] reported a learning curve of 40 cases for the DSA, and once surgical proficiency is achieved, the operative time is comparable to that of conventional surgery. Therefore, training of orthopedic surgeons should be enhanced to improve their proficiency in the DSA techniques. In addition, the results of this research index have high heterogeneity, and the reasons for the analysis may vary due to the different countries and regions included in the study, and the qualifications and experience of surgeons.

### Incision length

The size of the incision affects intraoperative blood loss and operative time, as well as increases the risk of injury to the vascular nerves, and a smaller incision allows for a faster recovery and a reduced hospital stay [25]. The results of this study showed that the DSA has significant advantages in terms of surgical incision compared to the conventional hip arthroplasty approach, and a total of 152 total hip arthroplasties with the DSA were included in the study by Eustathios and Feng Bin et al. [13, 15], with an average surgical incision < 10 cm. Such small incisions cause less damage to soft tissues and reduce the damage to peri-articular soft tissues of medical origin. For example, a direct anterior approach can cause lateral femoral cutaneous nerve injury [26], and a lateral approach by severing the gluteus minimus for access may lead to superior gluteal nerve injury [27]. In addition, the posterior lateral approach requires the dissection of the piriformis muscle to expose the joint capsule [28]. The DSA incision is minimally invasive unlike the traditional lateral and posterior lateral approaches, which preserve the iliotibial bundle, femoral square, and obturator externus tendon [13]. The rate of postoperative dislocation is reduced by not cutting the iliotibial bundle, the preservation of the short external rotators improves hip stability and functional outcomes [23, 29]. Phruethiphat et al. [30] proposed the piriformis-sparing approach for hemiarthroplasty in elderly patients with femoral neck fractures, and Hanly et al. [31] mentioned the SPAIRE approach for total hip arthroplasty. Both approaches preserve the piriformis muscle and achieve better outcomes, such as lower dislocation rates and faster recovery periods, but do not preserve tissues such as the iliotibial bundle and the distal short external rotators. However, there is a paucity of studies on the DSA versus the piriformis-sparing and SPAIRE approach, and more evidence is needed to illustrate the superiority of the DSA versus these two approaches in terms of hip dislocation and functional outcomes. Therefore, the DSA is beneficial for more rapid patient recovery because of smaller surgical incisions and less injury, thereby reducing the chance of postoperative pain and swelling caused by the incision and reducing postoperative complications.

### Intraoperative blood loss and blood transfusion rate

The amount of blood loss and transfusion rate in hip joint replacement surgery are closely related to intraoperative bleeding, such as osteotomy, medullary cavity expansion, soft tissue resection, and surgical time. Increased blood loss and transfusion rate can cause hypoalbuminemia and hypocalcemia, and the complications of hypoalbuminemia and hypocalcemia (such as muscle weakness, spasms, etc.) can increase the length of hospital stay, prolong the patient's recovery process, and even increase the chance of readmission [32]. The results of this study showed that the amount of blood loss and transfusion rate in total hip arthroplasty with DSA were lower than those in the control group. The DSA, as a new approach, although slightly longer in surgical time than the control group, showed significant superiority in terms of bleeding and transfusion rate. This may be related to the smaller soft tissue damage and smaller incision of the DSA. Eustathios et al. [15] and Matthew et al. [17] found that the DSA reduced intraoperative blood loss and transfusion rates by 8% and 2.6%, respectively, compared to the control group. Therefore, compared to traditional surgery, the DSA can reduce the incidence of complications such as hypoalbuminemia after total hip arthroplasty. Intraoperative blood loss is also an independent risk factor for transfusion after hip replacement surgery. Less bleeding reduces the transfusion rate after surgery. In addition, in situations where blood products are scarce, the DSA can allow for more effective use of blood due to its lower transfusion rate.

### Length of stay

The length of hospital stay can affect patients' physiological, psychological, and Social aspects. A shorter hospital stay can reduce the psychological and economic pressures caused by the disease [33, 34]. The results of this study show that the hospital stay of the DSA group is shorter than that of the traditional method group. H.J. Leonard et al.'s study [18] suggests that DSA is also superior to traditional total hip replacement in terms of postoperative physical rehabilitation time. Therefore, using the DSA technology for surgery significantly reduces the cost of total hip replacement. In addition, it also saves our limited medical resources to a certain extent, making medical resources used reasonably.

### Limb length differences and hip harris score

Differences in lower extremity length lead to compensatory and adaptive changes in certain musculoskeletal systems, starting with the psychemotional state of the patient and eventually leading to persistent anatomical and functional disturbances [35, 36]. But the overall impact depends on the cause and magnitude of the difference [37]. The results of this study showed that the traditional group was superior to the DSA group in terms of the difference in the length of the bilateral lower extremities. The reason for this result may be that TsiridisE et al. [38] found that DSA has problems such as exposure field of view, small range, and narrow operating space, which affects the judgment and control of the prosthesis placement angle. The difference in the length of the lateral lower extremity was slightly larger, but these differences had little effect on the function of the postoperative hip joint of the patients. The HHS was developed to assess the outcome of hip surgery to assess various hip disabilities and treatments in the adult population, with higher HHS indicating greater hip physiology [39, 40]. The results of this study showed that there was no significant difference in mid-term HHS scores between the two groups, but due to the short overall follow-up time, long-term follow-up evaluation is still needed for long-term efficacy. Eustathios et al. [14] collected the postoperative Harris hip joint data of 100 patients in the DSA group and found that the postoperative 1-month results of the DSA group were better than those of the traditional group. However, these long-term results require further research. In addition, in this study, only two studies with a total of 152 patients were included in the analysis, which may limit the accuracy of our judgment.

### Oxford hip score

The OHS score is a comprehensive marker widely used to evaluate the hip function and assess the outcome of hip arthroplasty [41]. The OHS is a validated self-administered questionnaire consisting of 12 items related to daily tasks directly affected by poor hip function, with higher scores indicating better hip function in the short term after hip replacement [42]. In this study, the results showed that the OHS of the DSA group was slightly better than that of the conventional surgery group 3 months after surgery. Therefore, the DSA has certain advantages in early postoperative recovery. At the 12-month follow-up, there was little difference between the two groups. Based on the above analysis, the DSA can significantly improve the early functional recovery of patients after surgery, and improve the satisfaction and acceptance of hip replacement. However, there is little difference between the DSA in terms of medium- and long-term safety and effectiveness. To seek a longer-term curative effect, further research is needed, as well as more clinical data support.

## Limitations

This article has some limitations: (1) the follow-up and some evaluation metrics were inconsistent across studies, (2) the analysis lacked detailed scoring data and did not assess complication rates, (3) only published studies were included, therefore, unpublished articles may affect the final results, (4) the DSA technique was first reported in 2012 and still requires a multicenter, larger sample of follow-up evaluations to determine its long-term efficacy and complications, (5) the limited literature included in this article and the small number of RCTs may affect the reliability of the results, larger sample sizes and well-designed RCTs are needed to confirm our conclusions, (6) In the current study, some of the parameters, such as operative time and incision length, had a high degree of heterogeneity, so their reliability was low.

## Foreground perspective

In actual clinical practice, this study provides an evidence-based basis for orthopedic surgeons to choose a surgical approach with few complications, rapid recovery, and minimal injury. The use of direct superior approach for total hip arthroplasty, without affecting the position of the acetabular prosthesis, can avoid muscle injury and obtain rapid recovery of hip function, which is a safe and reliable surgical access and is worthy of promotion and application. The description "Minimally Invasive Surgery" (MIS) is fuelling new interest among orthopedic surgeons in surgical access to the hip, and minimally invasive orthopaedics is more the future.

## Conclusions

The results of this study show that there is no significant difference in early hip-related scores between the DSA group and the conventional approach group after surgery. However, the DSA approach is superior to the traditional approach in terms of incision length, blood loss, postoperative transfusion rate, and early postoperative HHS. In addition, our study concludes that due to less tissue damage caused by DSA, it has potential advantages such as accelerating postoperative recovery, shortening hospital stay stays, and reducing patient economic pressure, which can significantly improve patient quality of life and satisfaction.

## Data Availability

All data generated or analyzed during this study are included in this published article [and its supplementary information files].
